# Supplementary figures and images for: Host tropism determination by convergent evolution of immunological evasion in the Lyme disease system
Source: PLoS Pathog. 2021 Jul 29;17(7):e1009801. doi: 10.1371/journal.ppat.1009801 (PMC8354441; doi:10.1371/journal.ppat.1009801)

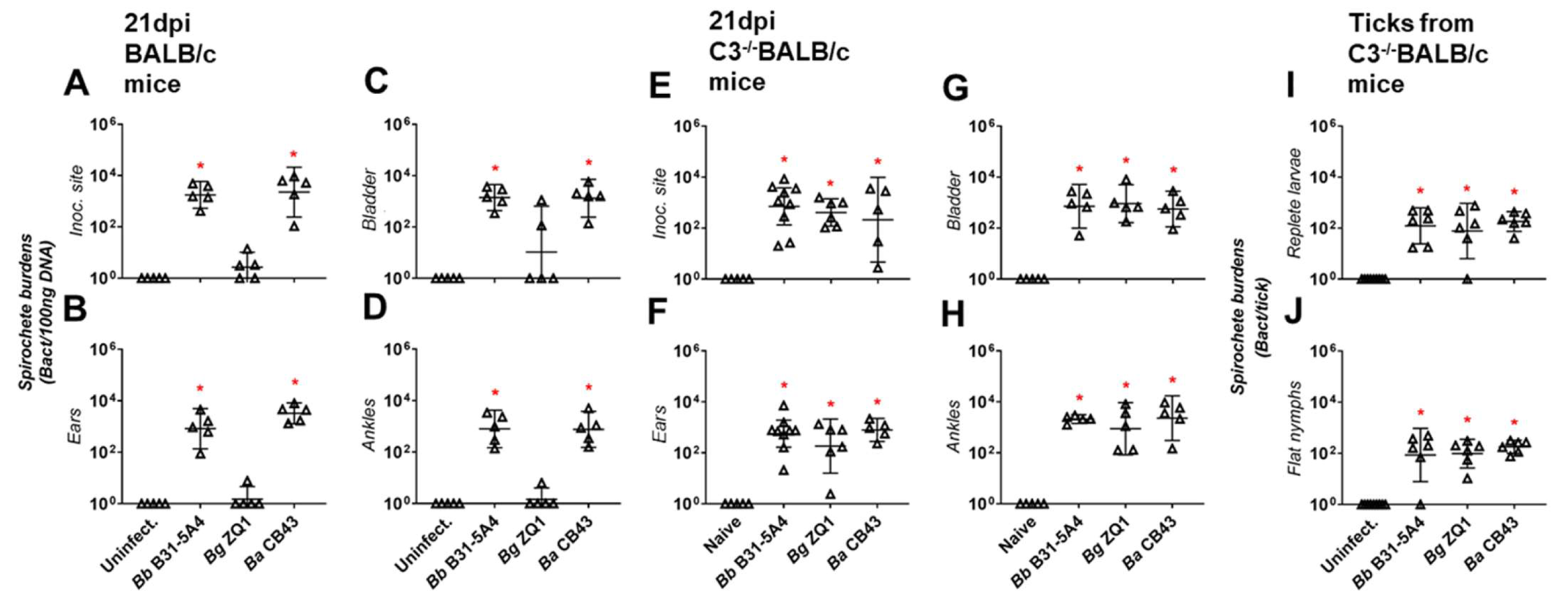

Supplement: S1 Fig — (A to D) BALB/c or (E to H) C3-/- BALB/c mice were injected with 106 B. burgdorferi B31-5A4 (“Bb B31-5A4”), B. garinii ZQ1 (“Bg ZQ1”), or B. afzelii CB43 (“Ba CB43”). At 21 days post injection (“dpi”), spirochete burdens were determined in the (A and E) inoculation site (“Inoc. site”), (B and F) ears, (C and G) bladder, (D and H) ankles. Additionally, uninfected I. scapularis larvae were allowed to feed on those C3-/- mice at 14 days post injection (“dpi”) to repletion. Spirochete burdens in (I) replete larvae, and (J) post molting flat nymphs (“flat nymphs”) were determined. The tissues from uninfected mice and uninfected nymphs were included as control (“Uninfect.”). Shown are the geometric means of bacterial loads ± 95% confidence interval of bacterial burdens from 6 replete larvae, flat nymphs or tissues from 5 BALB/c mice or indicated numbers of C3-/- BALB/c mice (9 Bb B31-5A4-infected inoculation sites and ears, 6 Bg ZQ1-infected inoculation sites and ears, or 5 all other tissues). Significant differences (p < 0.05, Kruskal-Wallis test with the two-stage step-up method of Benjamini, Krieger, and Yekutieli) in the spirochete burdens relative to uninfected ticks or tissues (*) are indicated. (TIF) [file ppat.1009801.s001.tif]

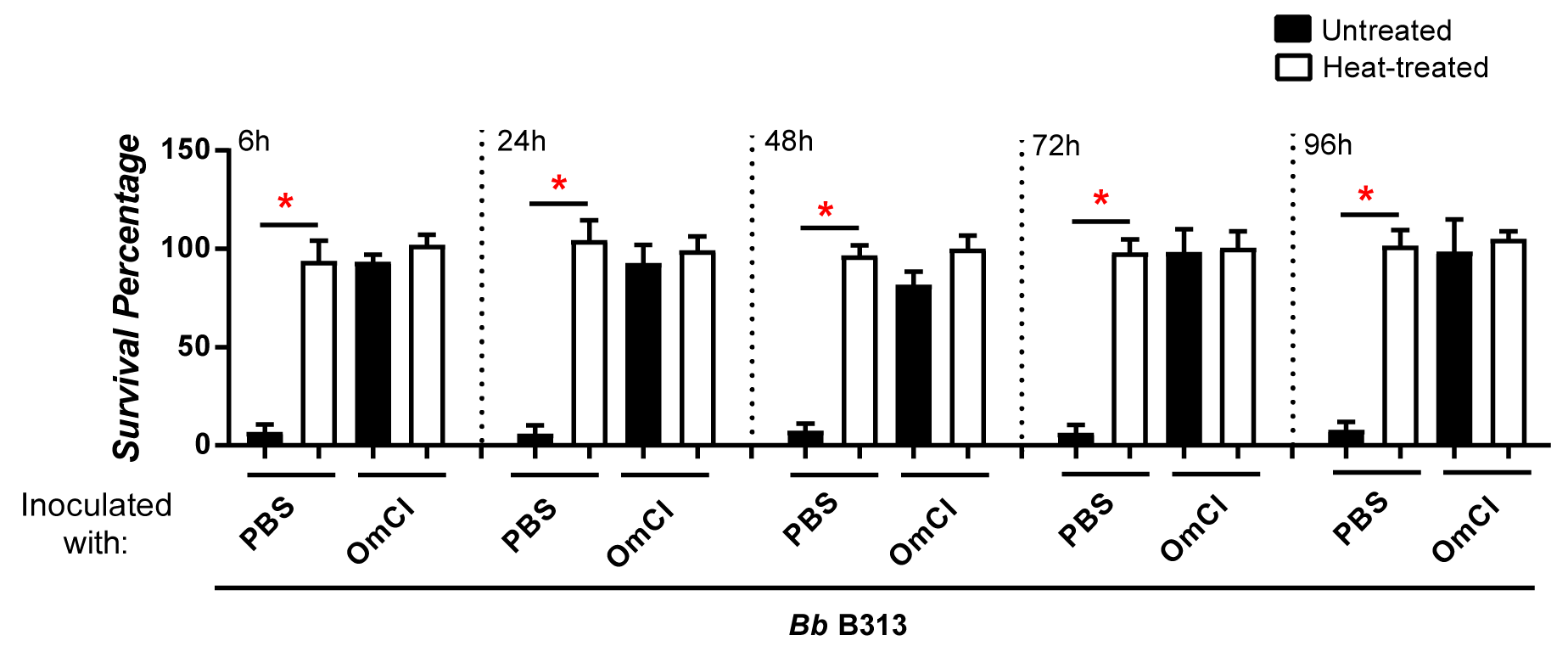

Supplement: S2 Fig — Coturnix quail were subcutaneously injected with OmCI (1 mg/kg of quail) or PBS buffer. Untreated (black bars) or heat-treated (white bars) of sera collected from these quail at indicated time points after inoculation were incubated with a serum-sensitive, highly passaged B. burgdorferi strain B313 for 0-h and 4-h with a final concentration of 40%. The number of motile spirochetes was assessed through microscopy. The survival percentage of the spirochetes was calculated using the number of mobile spirochetes at 4 h post incubation normalized to that at 0 h of incubation with serum. Each bar represents the mean of three independent determinations ± SEM from sera from five quail per group. Significant differences (p < 0.05, Kruskal-Wallis test with the two-stage step-up method of Benjamini, Krieger, and Yekutieli) in the percentage survival of spirochetes incubated with untreated sera from OmCI-inoculated quail, compared to that in heat-inactivated sera from those quail (*). (TIF) [file ppat.1009801.s002.tif]

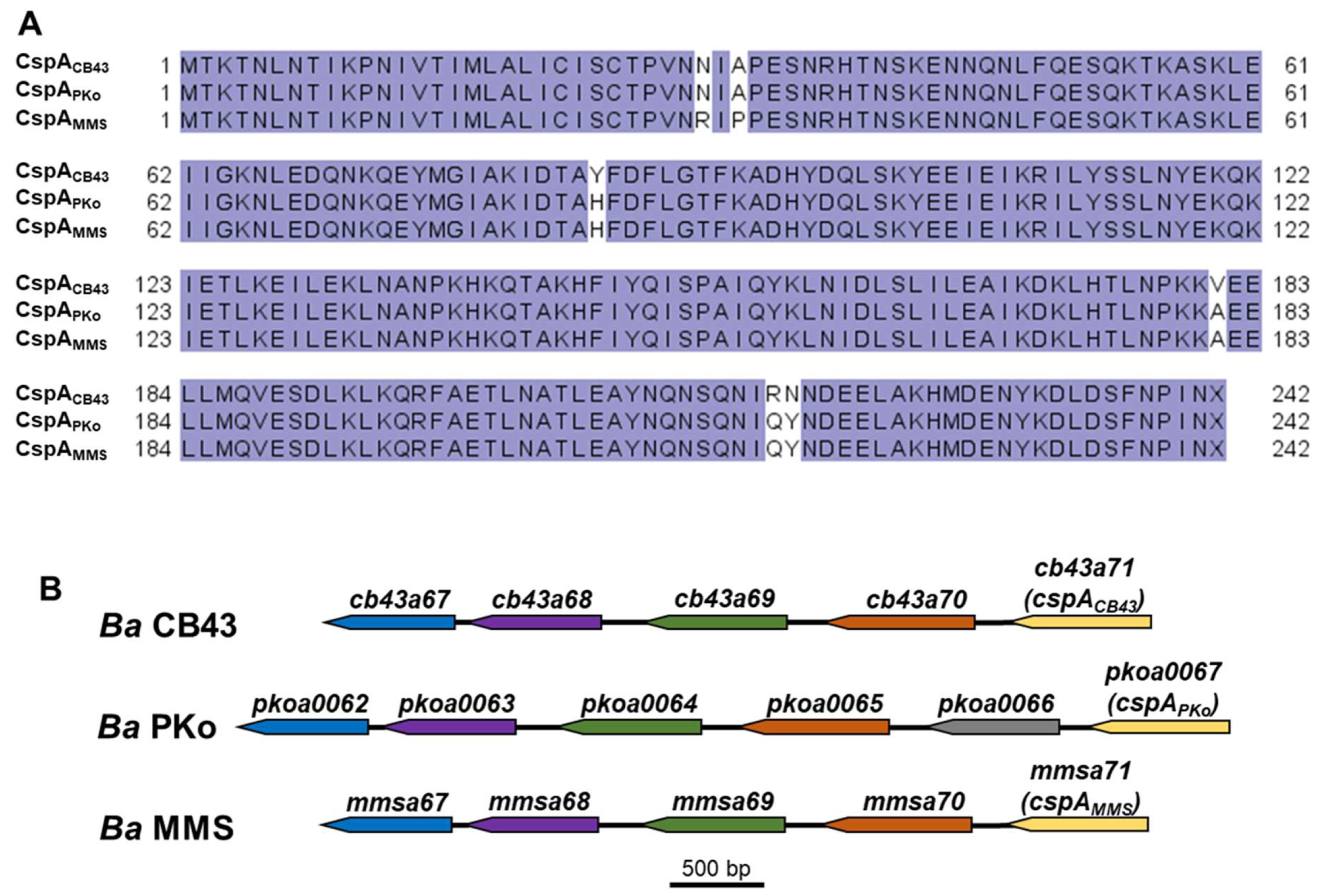

Supplement: S3 Fig — (A) CspA variants from B. afzelii CB43, PKo, and MMS aligned in M-Coffee. Blue shading indicates complete sequence conservation. (B) Synteny of Pfam54-IV genes. Colors indicate one-to-one orthologs. Locus pkoa0066 is only found in strain PKo. Scale bar denotes 500 bp. (TIF) [file ppat.1009801.s003.tif]

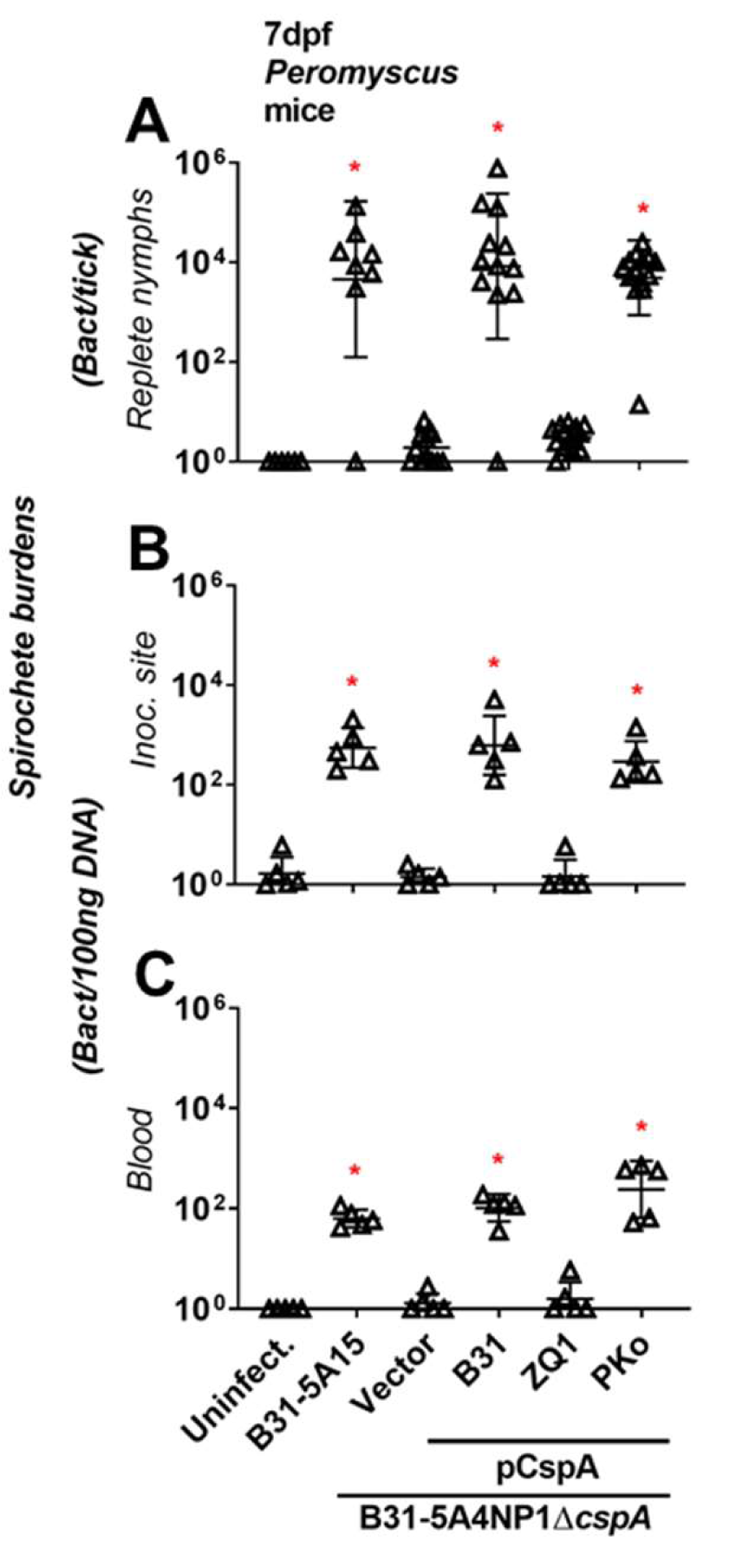

Supplement: S4 Fig — Ixodes scapularis nymphs infected by WT B. burgdorferi B31-5A15 (“B31-5A15”), B. burgdorferi B31-5A4NP1ΔcspA transformed with an empty shuttle vector (“Vector”), or this deletion strain producing a mutated variant of CspA from B. burgdorferi B31 selectively devoid of FH binding activity (“L246D”), WT B. burgdorferi B31 (“B31”), B. garinii ZQ1 (“ZQ1”), or B. afzelii PKo (“PKo”) were allowed to feed to repletion on P. leucopus mice. Uninfected nymphs and P. leucopus mouse tissues and blood were included as control (“Uninfect.”). Fed nymphs were collected upon repletion, and blood and tissues were collected at 7 days post nymph feeding (“dpf”). Spirochete burdens in (A) replete nymphs, (B) tick bite sites of skin (“Inoc. site”), and (C) blood were determined by qPCR. For spirochete burdens in tissue samples, the resulting values were normalized to 100 ng of total DNA. Shown are the geometric means of bacterial loads ± 95% confidence interval of bacterial burdens in tissues from five P. leucopus mice per group or replete nymphs (8 nymphs carrying the strain B31-5A15, 12 nymphs carrying the strain “Vector”, 13 nymphs carrying the strain pCspA-B31, 15 nymphs carrying the strain pCspA-PKo, or 13 nymphs carrying the strain pCspA-ZQ1). Significant differences (p < 0.05, Kruskal-Wallis test with the two-stage step-up method of Benjamini, Krieger, and Yekutieli) in the spirochete burdens relative to uninfected ticks or P. leucopus mouse tissues or blood are indicated (*). (TIF) [file ppat.1009801.s004.tif]

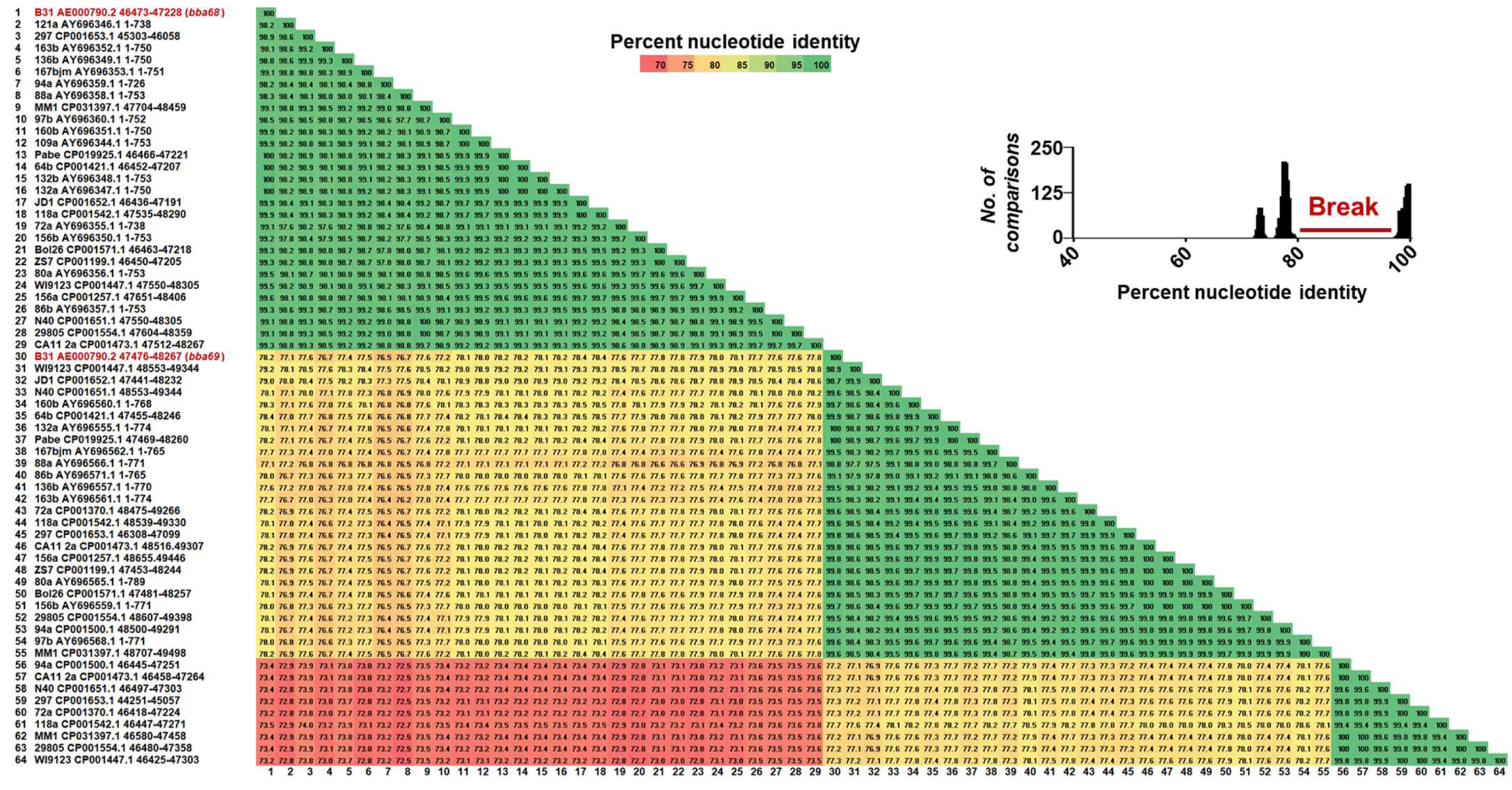

Supplement: S5 Fig — Nucleotide sequences encoding PFam54-IV proteins from B. burgdorferi B31 were used as queries to mine NCBI GenBank for orthologs. (inset) Frequency distribution of pairwise genetic distances. The pairwise identity numbers are coded by color gradually from identical (100% pairwise identity; green) to divergent sequences (70% pairwise identity; red). The clear break in the frequency distribution separates highly similar (> 95% pairwise identity) from moderately divergent comparisons (< 80%). The Pfam54-IV ortholog from a particular B. burgdorferi strain with > 95% identity to cspAB31 was defined as the cspA ortholog in that strain. (TIF) [file ppat.1009801.s005.tif]

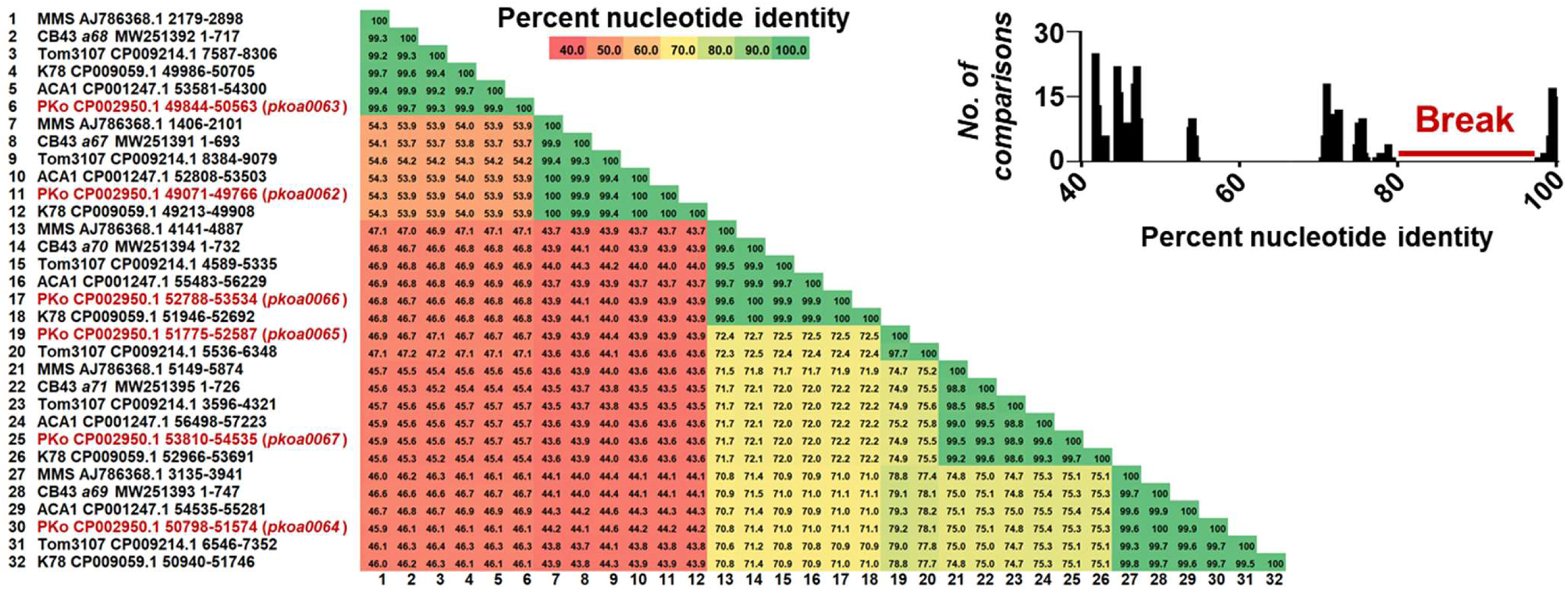

Supplement: S6 Fig — Nucleotide sequences encoding PFam54-IV proteins from B. afzelii PKo were used as queries to mine NCBI GenBank for orthologs. (inset) Frequency distribution of pairwise genetic distances. The pairwise identity numbers are coded by color gradually from identical (100% pairwise identity; green) to divergent sequences (40% pairwise identity; red). The clear break in the frequency distribution separates highly similar (> 98% pairwise identity) from moderately divergent comparisons (< 80%). The Pfam54-IV ortholog from a particular B. afzelii strain with > 98% identity to cspAB31 was defined as the cspA ortholog in that strain. (TIF) [file ppat.1009801.s006.tif]

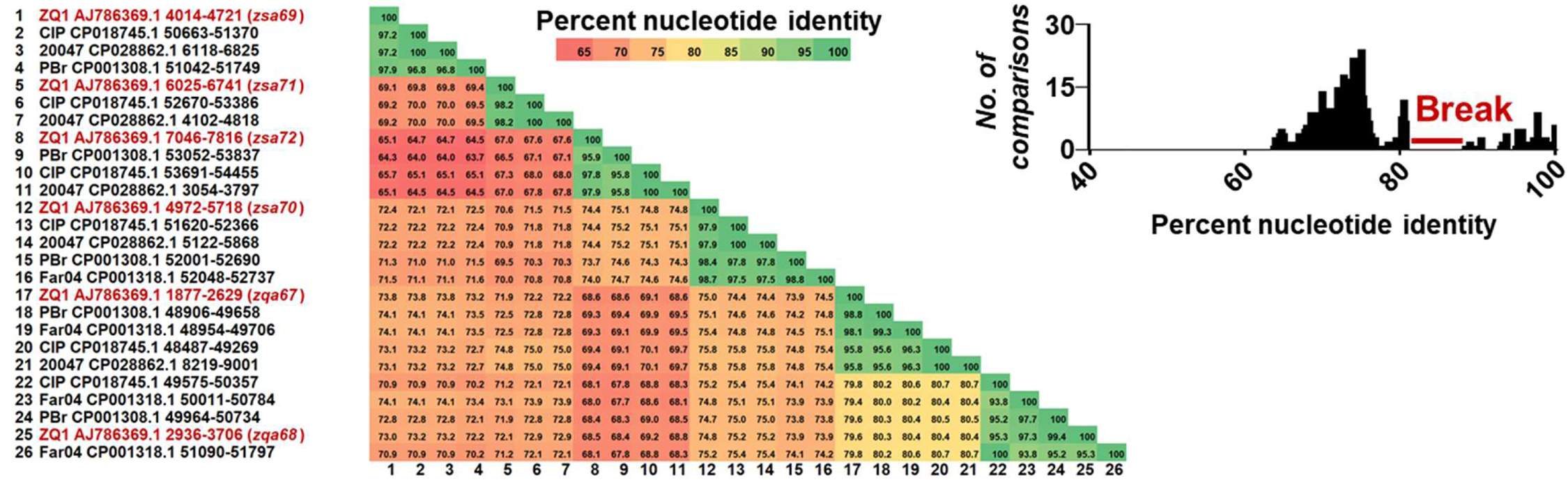

Supplement: S7 Fig — Nucleotide sequences encoding PFam54-IV proteins from B. garinii ZQ1 were used as queries to mine NCBI GenBank for orthologs. (inset) Frequency distribution of pairwise genetic distances. The pairwise identity numbers are coded by color gradually from identical (100% pairwise identity; green) to divergent sequences (65% pairwise identity; red). The clear break in the frequency distribution separates highly similar (> 93% pairwise identity) from moderately divergent comparisons (< 80%). The Pfam54-IV ortholog from a particular B. garinii strain with > 93% identity to cspAB31 was defined as the CspA ortholog in that strain. (TIF) [file ppat.1009801.s007.tif]
