# Supplementary material for: Host tropism determination by convergent evolution of immunological evasion in the Lyme disease system
Source: PLoS Pathog. 2021 Jul 29;17(7):e1009801. doi: 10.1371/journal.ppat.1009801 (PMC8354441; doi:10.1371/journal.ppat.1009801)
Supplement: S1 Table — (DOCX) [file ppat.1009801.s008.docx]

**S1 Table. Summarized findings of allelically variable, host-specific transmissibility and FH-binding activity of *B. burgdorferi*, *B. afzelii*, and *B. garinii* and their derived PFam54-IV proteins in this study and the previous study.**

| **Spirochete strains** | **Transmissibility by wild type strains^a^** | | **PFam54-IV variants** | **Transmissibility by Δ*cspA* producing PFam54-IV^c^** | | **FH-binding activity^f^** | |
| --- | --- | --- | --- | --- | --- | --- | --- |
|  | **Mouse** | **Quail** |  | **Mouse** | **Quail** | **Mouse** | **Quail** |
| ***B. burgdorferi* B31-5A4** | + | + | BBA68 | + | + | + | + |
|  |  |  | BBA69 | n.d.^d^ | n.d. | - | - |
|  | | | | | | | |
| ***B. afzelii***  **CB43,**  **PKo,**  **or MMS** | +^b^ | -^b^ | MMSA67 | n.d. | n.d. | - | - |
|  |  |  | MMSA68 | n.d. | n.d. | - | - |
|  |  |  | MMSA69 | n.d. | n.d. | - | - |
|  |  |  | MMSA70 | n.d. | n.d. | - | - |
|  |  |  | PKoA71 (CspA_PKo_)  or MMSA71 (CspA_MMS_) | +^e^ | -^e^ | +^g^ | -^g^ |
|  | | | | | | | |
| ***B. garinii***  **ZQ1** | - | + | ZQA67 | n.d. | n.d. | - | - |
|  |  |  | ZQA68 (CspA_ZQ1_) | - | + | - | + |
|  |  |  | ZSA69 | n.d. | n.d. | - | - |
|  |  |  | ZSA70 | n.d. | n.d. | - | - |
|  |  |  | ZSA71 | n.d. | n.d. | - | - |
|  |  |  | ZSA72 | n.d. | n.d. | - | - |

^a^Significant increase or no difference in spirochete burdens in indicated mouse or quail compared to uninfected respective animals is defined as “+” and “-”, respectively. The results were shown in Figure 1A to C and G to I.

^b^Determined using *B. afzelii* CB43

^c^Significant increase or no difference in spirochete burdens in indicated mouse or quail compared to uninfected respective animals is defined as “+” and “-”, respectively. The results are shown in Figure 3A to C and [1].

^d^Not determined.

^e^Determined using the *B. burgdorferi* strain Δ*cspA* harboring the plasmid producing PKoA71(CspA_PKo_).

^f^Determined by qualitative ELISA in Figure 6A to C and described in [1]. Statistically increasing or no different levels of binding by indicated PFam54-IV compared to that by negative control DbpA proteins are defined as “+” and “-.”

^g^Determined using recombinant version of MMSA71 (CspA_MMS_).

**REFERENCES**

1. Hart T, Nguyen NTT, Nowak NA, Zhang F, Linhardt RJ, Diuk-Wasser M, et al. Polymorphic factor H-binding activity of CspA protects Lyme borreliae from the host complement in feeding ticks to facilitate tick-to-host transmission. PLoS pathogens. 2018;14(5):e1007106.
