# Supplementary material for: Host tropism determination by convergent evolution of immunological evasion in the Lyme disease system
Source: PLoS Pathog. 2021 Jul 29;17(7):e1009801. doi: 10.1371/journal.ppat.1009801 (PMC8354441; doi:10.1371/journal.ppat.1009801)
Supplement: S2 Table — (DOCX) [file ppat.1009801.s009.docx]

**S2 Table. Bacteria strains, and plasmids used in this study**

| Strain or plasmid | Genotype or characteristic | Sources |
| --- | --- | --- |
| *Borrelia* strains |  |  |
| ZQ1 | Clonal isolate of *B. garinii* strain ZQ1 | [1] |
| PKo | Clonal isolate of *B. afzelii* strain PKo | [2] |
| CB43 | Clonal isolate of *B. afzelii* strain CB43 | [3] |
|  |  |  |
| B31-5A15 | Clonal isolate of *B. burgdorferi* strain B31 lacking lp21 | [4] |
| B31-5A4NP1Δ*cspA* | B31-5A4NP1, the clonal isolate of strain B31 with *bbe02*:: KanR^a^, *cspA*::StrR^b^ , and lacking lp21 | [5] |
| B31-5A4NP1Δ*cspA* /pBSV2G | B31-5A4NP1Δ*cspA* carrying plasmid pBSV2G | [6] |
| B31-5A4NP1Δ*cspA* /pBSV2G-CspA_B31_ | B31-5A4NP1Δ*cspA* complemented with intact *cspA* (*bba68*) from *B. burgdorferi* strain B31 under the control of *cspA* promoter from this strain (PcspA). | [6] |
| B31-5A4NP1Δ*cspA* /pBSV2G-CspA_PKo_ | B31-5A4NP1Δ*cspA* complemented with intact *cspA* (*bafPKo_A0067*) from *B. afzelii* strain PKo under the control of PcspA. | [6] |
| B31-5A4NP1Δ*cspA* /pBSV2G-CspA_ZQ1_ | B31-5A4NP1Δ*cspA* complemented with intact *cspA* (*zqa68*) from *B. garinii* strain ZQ1 under the control of PcspA. | [6] |
| B31-5A4NP1Δ*cspA* /pBSV2G-CspA_B31_L246D | B31-5A4NP1Δ*cspA* complemented with intact *cspA* from *B. burgdorferi* strain B31 with leucine-246 replaced by aspartate under the control of PcspA. | [6] |
|  |  |  |
| *E. coli* strains |  |  |
| DH5α | F- Φ80lacZΔM15 Δ(lacZYA-argF) U169 recA1 endA1 hsdR17(rk-, mk+) phoA supE44 thi-1 gyrA96 relA1 λ- | ThermoFisher |
| BL21 | F–, ompT, hsdSB (rB–, mB–), dcm, gal, λ(DE3) | Promega |
| M15 [Prep4] | F-, Φ80ΔlacM15, thi, lac-, mtl-, recA+ , KmR | Qiagen |
| BL21/pET30a-DbpA_B31_ | BL21 producing histidine tagged residue 26 to 192 of DbpA from *B. burgdorferi* strain B31 | [7] |
| M15 [Prep4]/pQE-CspA_B31_ | M15 producing histidine tagged residue 26 to 252 of CspA (BBA68) from *B. burgdorferi* strain B31 | [6] |
| M15 [Prep4]/pQE-BBA69 | M15 producing histidine tagged residue 26 to 264 of BBA69 from *B. burgdorferi* strain B31 | This study |
| M15 [Prep4]/pQE- MMSA67 | M15 producing histidine tagged residue 20 to 232 of MMA67 from *B. afzelii* strain MMS | This study |
| M15 [Prep4]/pQE- MMSA68 | M15 producing histidine tagged residue 20 to 240 of MMSA68 from *B. afzelii* strain MMS | This study |
| M15 [Prep4]/pQE- MMSA69 | M15 producing histidine tagged residue 23 to 269 of MMSA69 from *B. afzelii* strain MMS | This study |
| M15 [Prep4]/pQE- MMSA70 | M15 producing histidine tagged residue 26 to 239 of MMSA70 from *B. afzelii* strain MMS | This study |
| M15 [Prep4]/pQE-CspA_MMS_ | M15 producing histidine tagged residue 28 to 242 of CspA (MMSA71) from *B. afzelii* strain MMS | [8] |
| M15 [Prep4]/pQE-ZQA67 | M15 producing histidine tagged residue 26 to 251 of ZQA67 from *B. garinii* strain ZQ1 | This study |
| M15 [Prep4]/pQE-CspA_ZQ1_ | M15 producing histidine tagged residue 27 to 256 of CspA (ZQA68) from *B. garinii* strain ZQ1 | [8] |
| M15 [Prep4]/pQE-ZSA69 | M15 producing histidine tagged residue 26 to 236 of ZSA69 from *B. garinii* strain ZQ1 | This study |
| M15 [Prep4]/pQE-ZSA70 | M15 producing histidine tagged residue 26 to 249 of ZSA70 from *B. garinii* strain ZQ1 | This study |
| M15 [Prep4]/pQE-ZSA71 | M15 producing histidine tagged residue 26 to 239 of ZSA71 from *B. garinii* strain ZQ1 | This study |
| M15 [Prep4]/pQE-ZSA72 | M15 producing histidine tagged residue 25 to 257 of ZSA72 from *B. garinii* strain ZQ1 | This study |
|  |  |  |
| Plasmid |  |  |
| pJET1.2/Blunt | AmpR^c^; PCR cloning vector | ThermoFisher |
| pQE30Xa | AmpR^c^; histidine-tag protein expression vector | Qiagen |
| pQE-CspA_B31_ | pQE30Xa encoding histidine fusion protein residue 26 to 252 of CspA (BBA68) from *B. burgdorferi* strain B31 | [6] |
| pQE-BBA69 | pQE30Xa encoding histidine fusion protein residue 26 to 264 of BBA69 from *B. burgdorferi* strain B31 | This study |
| pQE-MMSA67 | pQE30Xa encoding histidine fusion protein residue 20 to 232 of MMA67 from *B. afzelii* strain MMS | This study |
| pQE-MMSA68 | pQE30Xa encoding histidine fusion protein residue 20 to 240 of MMSA68 from *B. afzelii* strain MMS | This study |
| pQE-MMSA69 | pQE30Xa encoding histidine fusion protein residue 23 to 269 of MMSA69 from *B. afzelii* strain MMS | This study |
| pQE-MMSA70 | pQE30Xa encoding histidine fusion protein residue 26 to 239 of MMSA70 from *B. afzelii* strain MMS | This study |
| pQE-CspA_MMS_ | pQE30Xa encoding histidine fusion protein residue 28 to 242 of CspA (MMSA71) from *B. afzelii* strain MMS | This study |
| pQE-ZQA67 | pQE30Xa encoding histidine tagged protein residue 26 to 251 of ZQA67 from *B. garinii* strain ZQ1 | This study |
| pQE-CspA_ZQ1_ | pQE30Xa encoding histidine tagged protein residue 27 to 256 of CspA (ZQA68) from *B. garinii* strain ZQ1 | [8] |
| pQE-ZSA69 | pQE30Xa encoding histidine tagged protein residue 26 to 236 of ZSA69 from *B. garinii* strain ZQ1 | This study |
| pQE-ZSA70 | pQE30Xa encoding histidine tagged protein residue 26 to 249 of ZSA70 of Zsa70 from *B. garinii* strain ZQ1 | This study |
| pQE-ZSA71 | pQE30Xa encoding histidine tagged protein residue 26 to 239 of ZSA71 from *B. garinii* strain ZQ1 | This study |
| pQE-ZSA72 | pQE30Xa encoding histidine tagged protein residue 25 to 257 of ZSA72 of Zsa72 from *B. garinii* strain ZQ1 | This study |
| pQE-CspA_B31_L246D | pQE30Xa encoding histidine fusion protein residue 26 to 251 of CspA (BBA68) from *B. burgdorferi* strain B31 with leucine-246 replaced by aspartate | [6] |
| pBSV2G | GenR^d^; pBSV2-derived shuttle vector. | [9] |
| pBSV2G-CspA_B31_ | pBSV2G encoding intact *cspA* (*bba68*) from *B. burgdorferi* strain B31 under the control of *cspA* promoter from this strain (PcspA). | [6] |
| pBSV2G-CspA_PKo_ | pBSV2G encoding intact *cspA* (*bafPKo_A0067*) from *B. afzelii* strain PKo under the control of pCspA | [6] |
| pBSV2G-CspA_ZQ1_ | pBSV2G encoding intact *cspA* (*zqa68*) from *B. garinii* strain ZQ1 under the control of pCspA | [6] |
| pBSV2G-CspA_B31_L246D | pBSV2G encoding intact *cspA* (*bba68*) from *B. burgdorferi* strain B31 under the control of pCspA with leucine-246 replaced by aspartate | [6] |

KanR^a^, Kanamycin resistant

StrR^b^, Streptomycin resistant

AmpR^c^, Ampicillin resistant

GenR^d^, Gentamicin resistant

**REFERENCES**

1. Kraiczy P, Hartmann K, Hellwage J, Skerka C, Kirschfink M, Brade V, et al. Immunological characterization of the complement regulator factor H-binding CRASP and Erp proteins of *Borrelia burgdorferi*. International journal of medical microbiology : IJMM. 2004;293 Suppl 37:152-7.

2. Hammerschmidt C, Koenigs A, Siegel C, Hallstrom T, Skerka C, Wallich R, et al. Versatile roles of CspA orthologs in complement inactivation of serum-resistant Lyme disease spirochetes. Infection and immunity. 2014;82(1):380-92.

3. Stepanova-Tresova G, Kopecky J, Kuthejlova M. Identification of *Borrelia burgdorferi* sensu stricto, *Borrelia garinii* and *Borrelia afzelii* in *Ixodes ricinus* ticks from southern Bohemia using monoclonal antibodies. Zentralblatt fur Bakteriologie : international journal of medical microbiology. 2000;289(8):797-806.

4. Purser JE, Norris SJ. Correlation between plasmid content and infectivity in *Borrelia burgdorferi*. Proceedings of the National Academy of Sciences of the United States of America. 2000;97(25):13865-70.

5. Kenedy MR, Vuppala SR, Siegel C, Kraiczy P, Akins DR. CspA-mediated binding of human factor H inhibits complement deposition and confers serum resistance in *Borrelia burgdorferi*. Infection and immunity. 2009;77(7):2773-82.

6. Hart T, Nguyen NTT, Nowak NA, Zhang F, Linhardt RJ, Diuk-Wasser M, et al. Polymorphic factor H-binding activity of CspA protects Lyme borreliae from the host complement in feeding ticks to facilitate tick-to-host transmission. PLoS pathogens. 2018;14(5):e1007106.

7. Benoit VM, Fischer JR, Lin YP, Parveen N, Leong JM. Allelic variation of the Lyme disease spirochete adhesin DbpA influences spirochetal binding to decorin, dermatan sulfate, and mammalian cells. Infection and immunity. 2011;79(9):3501-9.

8. Wallich R, Pattathu J, Kitiratschky V, Brenner C, Zipfel PF, Brade V, et al. Identification and functional characterization of complement regulator-acquiring surface protein 1 of the Lyme disease spirochetes *Borrelia afzelii* and *Borrelia garinii*. Infection and immunity. 2005;73(4):2351-9.

9. Tilly K, Krum JG, Bestor A, Jewett MW, Grimm D, Bueschel D, et al. *Borrelia burgdorferi* OspC protein required exclusively in a crucial early stage of mammalian infection. Infection and immunity. 2006;74(6):3554-64.
