# Supplementary material for: Host tropism determination by convergent evolution of immunological evasion in the Lyme disease system
Source: PLoS Pathog. 2021 Jul 29;17(7):e1009801. doi: 10.1371/journal.ppat.1009801 (PMC8354441; doi:10.1371/journal.ppat.1009801)
Supplement: S3 Table — (DOCX) [file ppat.1009801.s010.docx]

**S3 Table. Primers used in this study.**

| Primer/Vector | Sequence | Amplified DNA fragment |
| --- | --- | --- |
| 16srRNAfp | GCTTCGCTTGTAGATGAGTCTGC | *BB16srRNA* |
| 16srRNArp | TTCCAGTGTGACCGTTCACC |  |
| ColE1fp | CTACATACCTCGCTCTGCTAATC | *BBcolE1* |
| ColE1rp | CGAAACCCGACAGGACTATAAA |  |
| mNidfp | CCAGCCACAGAATCCCATCC | *mNidogen* |
| mNidrp | GGACATACTCTGCTGCCATC |  |
| Qβ-actinfp | CTGGCACCTAGCACAATGAA | *qβ-actin* |
| Qβ-actinrp | CTGCTTGCTGATCCACATCT |  |
| Kanfp | ATGAGCCATATTCAACGGGAA | Kanamycin |
| Kanrp | TTAGAAAAACTCATCGAGCAT |  |
| Genfp | ATGTTACGCAGCAGCAAC | Gentamycin |
| Genrp | TTAGGTGGCGGTACTTGG |  |
| Strfp | CAGGATGACGCCTAACAA | Streptomycin |
| Strrp | CCACCTTCAACAGATCGC |  |
